# Sex‐specific responses of bone metabolism and renal stone risk during bed rest

**DOI:** 10.14814/phy2.12119

**Published:** 2014-08-08

**Authors:** Jennifer L. L. Morgan, Martina Heer, Alan R. Hargens, Brandon R. Macias, Edgar K. Hudson, Linda C. Shackelford, Sara R. Zwart, Scott M. Smith

**Affiliations:** 1 Oak Ridge Associated Universities/NASA, NASA Johnson Space Center, Houston, Texas, USA; 2 Institute of Nutritional Physiology, University of Bonn, Bonn, Germany; 3 University of California San Diego, San Diego, California, USA; 4 JES Tech, NASA Johnson Space Center, Houston, Texas, USA; 5 NASA, NASA Johnson Space Center, Houston, Texas, USA; 6 Universities Space Research Association, Houston, Texas, USA

**Keywords:** Bed rest, bone density, bone metabolism, renal stone risk, sex differences

## Abstract

The purpose of this study was to directly assess sex differences in bone loss, bone biochemistry, and renal stone risk in bed rest. Bed rest simulates some spaceflight effects on human physiology and can be used to address the potential existence of sex‐specific effects on bone metabolism and renal stone risk in space. We combined data from the control subjects in five head‐down‐tilt bed rest studies (combined *n *= 50 men, 24 women) of differing durations (14–90 days). All subjects were healthy volunteers. Mean age was 35 ± 9 years for women and 33 ± 8 years for men. The main outcome measures were bone density and biochemistry, and renal stone risk chemistry. Before bed rest began, men had higher bone mineral density and content (*P* < 0.001), and excreted more biomarkers of bone resorption and calcium per day than did women (*P* < 0.05). These differences remained during bed rest. A number of urine chemistry analytes increased (e.g., calcium) or decreased (e.g., sodium, citrate, and urine volume) significantly for men and women during bed rest. These changes may predispose men to higher stone risk. Men and women do not have substantially different responses to the skeletal unloading of bed rest.

## Abstract (precis)

We report here that bed rest‐induced bone loss, bone biochemistry, and renal stone risk in humans does not depend on sex. These data are a compilation and analysis of original data from our multiple (published) studies; none of those were adequately powered to assess sex differences. Thus, the resulting findings provide an important new look at the data.

## Introduction

Long‐duration spaceflight is known to cause a wide variety of physiological changes, including changes in bone metabolism that result in bone loss. Bone loss on Earth occurs at disproportionate rates depending on a person's sex, particularly in the elderly population (Kanis et al. 2008). The existence of these sex‐specific effects during spaceflight has yet to be investigated and may have a large impact on astronaut selection. The long‐term bed rest model allows simulation of some of the physiological changes that occur during spaceflight (LeBlanc et al. 2007; Pavy‐Le Traon et al. 2007; Spector et al. 2009). The bed rest model provides several advantages over flight studies, including a larger subject pool and more extensive and rapid data collection. Despite these advantages, the number of bed rest subjects is still typically limited because of budget and other resource limitations, and few if any studies to date have been adequately powered to evaluate sex‐specific responses. Of the available published studies including either men or women, findings suggest that the response of bone to bed rest is similar for the two sexes (Smith et al. 2003; Zwart et al. 2007). However, these (typically single‐sex) studies do not allow a direct comparison of the responses of men and women to bed rest. Studies involving both sexes are typically not powered or designed to address sex‐specific questions. For one such example, in a 90‐day bed rest study with five women and eight men, sex and bed rest duration were analyzed for effects on bone markers, and no significant differences were found (Zwart et al. 2009b). Sex‐specific response was not a primary objective of that study, and it remains difficult to make definitive conclusions about sex effects on bone during bed rest.

Bone demineralization during spaceflight and bed rest increases urinary calcium, which alters urine chemistry to favor renal stone formation (Whitson et al. 1993, 1997, 1999; Monga et al. 2006; Okada et al. 2008). Renal stone risk, typically assessed by calculating relative urine supersaturation, is generally low during bed rest because subjects are encouraged to consume large amounts of fluid, specifically to minimize this risk. In the study mentioned above, only urinary sulfate was found to be affected by both bed rest and sex, but the report does not describe the effect further (Zwart et al. 2009b). Most other variables that contribute to renal stone risk, including urine volume, phosphorus, and pH, were different between the sexes, but any changes observed during bed rest were the same for men and women (Zwart et al. 2009b).

We sought here to combine data from several bed rest studies to determine whether differences in response to bed rest between men and women exist in bone mineral density, bone metabolism, and renal stone risk during bed rest. This approach allows the use of data from samples collected from a larger subject population, analyzed by the same laboratory.

## Methods

Data from five 6° head‐down‐tilt bed rest studies were combined and analyzed as a single data set. In total, 74 healthy subjects (24 women, 50 men) participated in one of the five bed rest studies. Sixteen subjects (four women, 12 men) participated in the 14‐day study and 12 subjects (four women, eight men) participated in the 30‐day study at the University of Texas Medical Branch (UTMB) (Morgan et al. 2012a,b); 13 subjects (seven women, eight men) participated in the 30‐day study at the University of California San Diego (UCSD) (Smith et al. 2003; Macias et al. 2007; Zwart et al. 2007); 18 subjects (six women, 12 men) participated in the 60‐day study at UTMB (Spector et al. 2009; Zwart et al. 2009b, 2010; Smith et al. 2012a) and 15 subjects (five women, 10 men) participated in the 90‐day study at UTMB (Spector et al. 2009; Zwart et al. 2009b, 2010; Smith et al. 2012a). All bed rest protocols complied with the World Medical Association Declaration of Helsinki – Ethical Principles for Medical Research Involving Human Subjects, and were reviewed and approved by the National Aeronautics and Space Administration (NASA) Johnson Space Center Institutional Review Board (IRB) and either the University of Texas Medical Branch (UTMB) IRB or the University of California San Diego (UCSD) IRB. All subjects received verbal and written explanation of the protocol and provided written informed consent.

**Figure 1.**
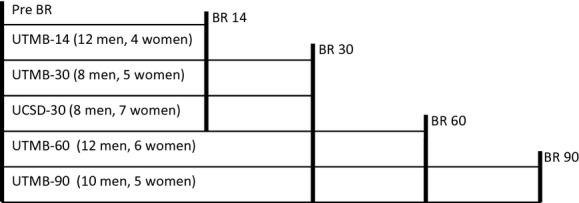
Graphic display of the multiple studies included here, along with timing of sample collection for blood and for 48‐h urine samples (indicated by vertical lines). Actual bed rest duration was used for each sample instead of the approximate values displayed in the scheme. BR, Bed rest (*day*); UTMB, bed rest study performed at University of Texas Medical Branch (Smith et al. 2008; Zwart et al. 2009b; Morgan et al. 2012a,b); UCSD, bed rest study performed at University of California San Diego (Macias et al. 2007; Zwart et al. 2007).

Only sedentary (i.e., control) subjects from these studies were used for this analysis. All bed rest protocols consisted of a 2‐ to 13‐day pre‐bed rest ambulation period followed by about 14, 30, 60, or 90 days of strict bed rest. The sampling times from each study used for this analysis are shown in Figure 1. All subjects were healthy and had no personal history of nephrolithiasis. Oral contraceptives were not used during the studies. The mean (±SD) age of the subjects was 35 ± 9 years for women and 33 ± 8 years for men, the mean weight was 63.4 ± 12.2 kg for women and 77.7 ± 11.4 kg for men, the mean height was 163.1 ± 7.5 cm for women and 175.3 ± 7.8 cm for men, and the mean body mass index was 23.7 ± 3.7 kg/m^2^ for women and 25.2 ± 2.9 kg/m^2^ for men.

The study methods have been previously described in detail (Meck et al. 2009; Spector et al. 2009; Zwart et al. 2009b, 2010; Smith et al. 2012a) and most of the data from each stand‐alone study have been presented previously (Smith et al. 2003; Zwart et al. 2007, 2009b; Morgan et al. 2012b), but none of the studies or publications had the primary objective of addressing possible differences between sexes. Although study designs, and sample and data collection procedures, may have varied from study to study (especially between the four studies conducted at UTMB and the one at UCSD), the data analyses were based on individual changes during bed rest. No subjects exercised or were treated with other countermeasures.

### Sample collection and biochemical analyses

A blood sample and two 24‐h urine samples were collected 1–3 days before bed rest and were the closest samples to the start of bed rest. Blood and two 24‐h urine samples were also collected just before the end of bed rest. Two 24‐h urine samples were not collected before reambulation for three subjects (two male, one female) in the 90‐day study or for seven subjects (six male, one female) in the 60‐day study for various unforeseen reasons including a hurricane evacuation and patient refusal. In addition to the pre‐bed rest and end of bed rest sample collections, samples were collected during bed rest for the 30‐, 60‐, and 90‐day studies (see Fig. 1 for additional sample collection days for each study, indicated by vertical lines). All samples were stored at −80°C until analysis. Analyses for each study were typically conducted in batch analysis within a matter of weeks of study (or in some cases, subject) completion. Samples were analyzed as part of the original study and the data were used for the analyses reported herein.

All blood and urine samples were analyzed in the Nutritional Biochemistry Laboratory at Johnson Space Center. This team has extensive experience with these types of sample collections, sample handling, and analyses. The laboratory is ISO Certified and participates in a number of external proficiency testing programs, including American Proficiency Institute (API), College of American Pathologists (CAP), Vitamin D External Quality Assessment Scheme (DEQAS), and National Institute of Standards and Technology (NIST), as detailed per test below. All analyses (detailed below) were run in duplicate, and had to meet assay specifications (e.g., within the assayed standard curve, and with high‐ and low‐level quality control samples having met laboratory guidelines for run acceptability).

Blood and urine samples were analyzed for indices of bone and calcium metabolism and vitamin D status, and have been previously described in detail (Smith et al. 2003, 2005a, 2008, 2009b, 2012a, 2014). Briefly, serum bone‐specific alkaline phosphatase (BSAP) was evaluated using commercially available kits (MicroVue^™^ BAP Kit, Quidel, San Diego, CA, 4.56% within‐assay and 7.79% between‐assay coefficient of variation [CV], CAP proficiency testing) (Smith et al. 2005b, 2008, 2009b, 2012b, 2014; Zwart et al. 2009b, 2011a). Total alkaline phosphatase was measured by colorimetry on an Olympus AU480^®^ analyzer (Beckman Coulter, Brea, CA, 0.9% within‐ and 2.6% between‐assay CV, API proficiency testing) (Smith et al. 2005b, 2008, 2009b, 2012b, 2014; Zwart et al. 2009b, 2011a). Osteocalcin was measured using commercial RIA kits (Biomedical Technologies, Stoughton, MA, 9% CV) as previously described (Zwart et al. 2011a). Undercarboxylated osteocalcin was determined using the same RIA, with an established method for quantifying the undercarboxylated fraction (Gundberg et al. 1998) that we have implemented previously (Zwart et al. 2011a). Assay CVs were 5.6% within and 5.7% between assay for osteocalcin and 4.3% within and 10.4% between assay for undercarboxylated osteocalcin. Total alkaline phosphatase (0.9% CV) was determined enzymatically using an Olympus AU480 analyzer. Vitamin D metabolites were determined by RIA (DiaSorin, Stillwater, MN). Assay CVs were 4.78% within and 13.25% between assay for serum 25‐hydroxyvitamin D and 7.46% within and 15.20% between assay for 1,25‐dihydroxyvitamin D. Proficiency testing was conducted with DEQAS, CAP, API, and NIST for 25‐hydroxyvitamin D and with DEQAS and CAP for 1,25‐dihydroxyvitamin D as described previously (Smith et al. 2005a,b, 2008, 2009a, 2012b; Zwart et al. 2011b, 2013).

Several biomarkers of collagen degradation were analyzed, including N‐telopeptide (NTX, 5.0% CV), C‐telopeptide (CTX, 6.9% CV), pyridinium cross‐links (PYD, 10.3% CV), deoxypyridinoline (DPD, 14.4% CV), and helical peptide (HP, 8.1% CV). All were analyzed using commercially available kits (NTX, Osteomark^®^ ELISA kit, Ostex International, Seattle, WA; CTX, Urine CrossLaps EIA, IDS, Bolton, Tyne & Wear, NE35 9PD, UK; PYD and DPD, Pyrilinks^™^ and Pyrilinks^™^‐D, respectively, Quidel, Santa Clara, CA; HP, MicroVue^™^ Helical Peptide EIA, Quidel), as described previously (Smith et al. 1999, 2003, 2004b, 2005a,b, 2008, 2009b, 2014). Serum total calcium and urinary calcium were determined by atomic absorption spectrophotometry (PerkinElmer, Waltham, MA, 1.86% within‐ and 2.52% between‐assay CVs, CAP proficiency testing) (Smith et al. 2005a,b, 2008, 2009b, 2012b,c, 2014; Zwart et al. 2009b, 2011a), and whole‐blood ionized calcium (<1% CV) was determined using a portable analyzer (i‐STAT^®^1 Analyzer, i‐STAT, East Windsor, NJ; between‐assay CV for ionized Ca = 1.15%) (Smith et al. 1997, 2004a). Urinary creatinine (5.1% CV) was analyzed using a colorimetric technique on an ACE Alera^®^ analyzer (Alfa Wassermann, West Caldwell, NJ, 2.99% within‐ and 3.30% between‐ assay CV, CAP proficiency testing) (Smith et al. 2005a,b, 2008, 2009b, 2012b, 2014; Zwart et al. 2009b, 2011a). Uric acid (0.95% CV) was measured by colorimetric assay on the Olympus AU480 auto analyzer (Beckman Coulter). Sodium (0.47% CV), magnesium (1.3% CV), and phosphorus (3.5% CV) were measured by ion‐selective assay on the Olympus AU480. Citrate (1.9% CV), sulfate (1.8% CV), and oxalate (7.0% CV), separately, were measured using a Dionex Ion Chromatography System 3000 (ICS‐3000) (Thermo Fisher Scientific, Sunnyvale, CA). The pH of urine samples was determined by potentiometry (Thermo Electron, Beverly, MA, 0.29% within‐ and 0.24% between‐assay CV, CAP proficiency testing) (Smith et al. 2005a, 2008, 2012b; Zwart et al. 2009a; Morgan et al. 2012b).

All samples from all of the studies were analyzed in the same laboratory. Some methods changed over time given the spread of study dates, but changes that occurred are described below and the new methods were fully validated and matched results from the old methods. Specifically, for intact PTH, for some subjects (*n *= 13), the samples were analyzed using the Nichols RIA (Nichols Institute Diagnostics, San Juan Capistrano, CA). For other subjects (*n *= 61), samples were analyzed using an immunoradiometric assay (Scantibodies, Santee, CA; 5.6% CV).

### Bone densitometry

As previously described (Smith et al. 2005b, 2008; Sibonga et al. 2007), areal bone mineral density (BMD) scans for both the UCSD and UTMB bed rest studies were performed by dual‐energy X‐ray absorptiometry (DXA) using either a Hologic QDR 450 or Hologic Discovery instrument (Hologic, Bedford, MA) at UTMB (Spector et al. 2009), or a Hologic Delphi W or Lunar DPX‐IQ instrument at UCSD (Zwart et al. 2007). Scans were performed 8–13 days before bed rest and again 1–2 days after bed rest, only on the subjects who participated in the 30‐day study at UCSD and the 60‐ and 90‐day studies at UTMB. DXA scans for each subject were performed by the same technician at each location.

### Dietary intake

As previously described for both studies (Zwart et al. 2007; Inniss et al. 2009), menus from ambulatory and bed rest phases of the study were prepared in a metabolic kitchen where all foods were weighed before they were consumed. Menus were rotated on an 11‐day cycle. Each subject's caloric intake was adjusted as necessary to maintain body weight within 3% of the weight measured on *day 3* of bed rest, and as a result, nutrient intakes were lower during bed rest than before (or after) bed rest. Actual dietary intakes were calculated using the Minnesota Nutrition Data System for Research (NDS‐R) software, version 5.0_35, May 2004, 2005, 2006, developed by the Nutrition Coordinating Center, University of Minnesota, Minneapolis, MN (Schakel et al. 1988).

### Statistical analysis

All statistical analyses were performed using Stata IC software (v 12.1, StataCorp, College Station, TX) and setting 2‐tailed *α* to reject the null hypothesis at *P* < 0.05. Transformation or exclusion of statistical outliers was required for some of our dependent variables, and these are indicated in the table footnotes. Separate mixed‐effects linear regression models (a.k.a. multi‐level models) were used to evaluate the effects of bed rest on our continuously scaled outcomes. For each outcome, our statistical model included dummy‐coded beta coefficients comparing the data from immediately before bed rest with all other time periods in a continuous linear fashion. As is typical with mixed‐effects modeling, all models included a random intercept to accommodate the longitudinal (within‐subject, repeated measures) experiment design (Goldstein 1995). Upon completion of these statistical models, the uncorrected *P*‐values were submitted to a single Bonferroni multiple‐testing correction. All tables report unadjusted *P*‐values. All *P*‐values <0.001 were below this Bonferroni‐adjusted critical *P* value of *P* < 0.0004.

## Results

Men generally had significantly greater BMD and bone mineral content (BMC) at most locations (Table 1 and Table 2, respectively). The exceptions were left and right hip neck BMD, left and right hip trochanter BMD, and total lumbar spine BMD, which were statistically not different between men and women. The response to bed rest was the same for men and women, leading to a loss of total BMC, pelvis BMD, left and right hip trochanter BMD, and left and right hip total BMD. The only statistically significant interactions between bed rest and sex were at the left and right hip neck BMD, where a decrease (−3% to −11%) in BMD occurred in women but an increase (+3%) occurred in men.

**Table 1.** Bone densitometry data before and after different durations of bed rest

Test	Bed rest duration, days					Effect		
	0	14	30	60	90	Sex	Duration	Interaction
*n*								
Women	18	0	7	6	5			
Men	26	0	4	12	10			
Total BMD, g/cm^2^								
Women	1.10 ± 0.06	ND	1.09 ± 0.04	1.12 ± 0.07	1.08 ± 0.05	<0.001		
Men	1.23 ± 0.10	ND	1.23 ± 0.11	1.19 ± 0.10	1.28 ± 0.08			
Pelvis BMD, g/cm^2^								
Women	1.11 ± 0.09	ND	1.11 ± 0.09	1.10 ± 0.10	1.06 ± 0.07	<0.01	<0.01	
Men	1.21 ± 0.12	ND	1.18 ± 0.16	1.14 ± 0.10	1.22 ± 0.11			
L hip neck BMD, g/cm^2^								
Women	0.86 ± 0.13	ND	0.97 ± 0.12^2^	0.83 ± 0.11	0.76 ± 0.08			<0.05
Men	0.89 ± 0.14	ND	0.93^2^	0.84 ± 0.12	0.92 ± 0.17			
R hip neck BMD,^1^ g/cm^2^								
Women	0.83 ± 0.10	ND	ND	0.85 ± 0.10	0.80 ± 0.10			<0.05
Men	0.90 ± 0.15	ND	ND	0.85 ± 0.11	0.93 ± 0.18			
L hip trochanter BMD, g/cm^2^								
Women	0.71 ± 0.10	ND	0.75 ± 0.10^2^	0.70 ± 0.11	0.64 ± 0.06		<0.001	
Men	0.77 ± 0.12	ND	0.77^2^	0.70 ± 0.08	0.80 ± 0.15			
R hip trochanter BMD,^1^ g/cm^2^								
Women	0.71 ± 0.08	ND	ND	0.70 ± 0.11	0.67 ± 0.03		<0.001	
Men	0.78 ± 0.12	ND	ND	0.72 ± 0.08	0.81 ± 0.14			
Total L hip BMD, g/cm^2^								
Women	0.94 ± 0.09	ND	0.96 ± 0.07^2^	0.95 ± 0.10	0.84 ± 0.09	<0.05	<0.001	
Men	1.04 ± 0.13	ND	0.98*	0.97 ± 0.09	1.07 ± 0.16			
Total R hip BMD,^1^ g/cm^2^								
Women	0.94 ± 0.10	ND	ND	0.96 ± 0.11	0.88 ± 0.09	<0.05	<0.001	
Men	1.04 ± 0.13	ND	ND	0.97 ± 0.08	1.08 ± 0.16			
Total lumbar spine BMD, g/cm^2^								
Women	1.05 ± 0.10	ND	1.16 ± 0.09	0.99 ± 0.06	1.00 ± 0.09			
Men	1.07 ± 0.12	ND	1.20 ± 0.20	0.99 ± 0.08	1.06 ± 0.08			

Values are means ±SD. BMD, bone mineral density; L, left; R, right; ND, not determined.

^1^ Test was not performed in the UCSD study.

^2^ Three male subjects and one female subject were removed at duration 30 because of DEXA scan issues.

**Table 2.** Bone mineral content and body composition before and after different durations of bed rest

Test	Bed rest duration, days					Effect		
	0	14	30	60	90	Sex	Duration	Interaction
*n*								
Women	18	0	7	6	5			
Men	26	0	4	12	10			
Total BMC,^1^ g								
Women	2126 ± 196	ND	ND	2134 ± 201	2059 ± 191	<0.001	<0.01	
Men	2819 ± 482	ND	ND	2573 ± 398	3064 ± 421			
Pelvis BMC,^1^ g								
Women	222 ± 26	ND	ND	216 ± 29	211 ± 18	<0.01		
Men	277 ± 70	ND	ND	241 ± 53	304 ± 60			
Total lumbar spine BMC,^1^ g								
Women	59 ± 6	ND	ND	55 ± 5	62 ± 6	<0.01		
Men	70 ± 12	ND	ND	64 ± 10	76 ± 10			
Total mass,^1^ kg								
Women	60.2 ± 10.9	ND	ND	61.7 ± 9.4	56.8 ± 11.5	<0.001		
Men	80.5 ± 11.2	ND	ND	75.5 ± 10.4	85.2 ± 11.2			
Total lean,^1^ kg								
Women	37.4 ± 4.5	ND	ND	36.4 ± 4.4	35.7 ± 4.1	<0.001	<0.01	
Men	56.5 ± 8.2	ND	ND	51.4 ± 6.1	60.2 ± 7.6			
Total lean,^1^ % lean								
Women	63 ± 6	ND	ND	59 ± 5	64 ± 6			
Men	70 ± 6	ND	ND	68 ± 5	71 ± 6			
Total fat,^1^ kg								
Women	20.8 ± 6.8	ND	ND	23.2 ± 6.3	19.5 ± 7.4		<0.05	
Men	21.2 ± 6.5	ND	ND	21.6 ± 6.4	22.0 ± 6.9			
Total fat,^1^ % fat								
Women	34 ± 6	ND	ND	37 ± 6	33 ± 6	<0.001	<0.001	
Men	26 ± 6	ND	ND	28 ± 6	25 ± 6			

BMC, bone mineral content; ND, not determined.

^1^ Test was not performed in the UCSD study.

Women had a greater percentage of body fat, and men had more total lean mass (kg) (Table 2). Total fat and percent fat increased whereas total lean mass decreased for both sexes during bed rest. No changes were detected in vitamin D metabolites during bed rest (Table 3). The bone formation markers BSAP and total alkaline phosphatase (ALP) were higher in men before bed rest. These markers increased significantly over the course of bed rest for both men and women. There was no difference in osteocalcin or undercarboxylated osteocalcin between men and women. Osteocalcin was unchanged during bed rest while undercarboxylated osteocalcin increased in both men and women. Serum calcium concentrations were higher (*P* < 0.01) in men than women but did not change during bed rest.

**Table 3.** Serum markers of bone and calcium metabolism before and during different durations of bed rest

Test	Outliers, no.	Bed rest duration, days					Effect		
		0	14	30	60	90	Sex	Duration	Interaction
*n*									
Women		24	13	20	11	5			
Men		50	29	37	22	10			
Bone‐specific alkaline phosphatase,^1^ U/L									
Women	0	21 ± 6	22 ± 6	22 ± 5	23 ± 4	27 ± 3	<0.01	<0.01	
Men		26 ± 7	27 ± 7	26 ± 6	26 ± 5	30 ± 4			
Alkaline phosphatase, U/L									
Women	1	51 ± 15	49 ± 16	56 ± 13	59 ± 10	70 ± 7	<0.05	<0.001	
Men		58 ± 12	62 ± 10	63 ± 12	67 ± 11	69 ± 10			
Osteocalcin,^1^ ng/mL									
Women	4	12 ± 4	11 ± 3	12 ± 3	12 ± 4	14 ± 6			
Men		12 ± 4	12 ± 4	14 ± 4	13 ± 3	13 ± 3			
Undercarboxylated osteocalcin,^1^^,^^2^ %									
Women	7	32 ± 8	34 ± 8	34 ± 6	39 ± 9	46 ± 2		<0.01	
Men		31 ± 8	30 ± 5	36 ± 5	39 ± 8	32 ± 13			
Parathyroid hormone,^1^ pg/mL									
Women	0	25 ± 10	18 ± 3	19 ± 7	23 ± 12	34 ± 17			
Men		22 ± 10	17 ± 7	19 ± 8	21 ± 10	23 ± 12			
25‐hydroxyvitamin D,^1^ nmol/L									
Women	1	59 ± 31	61 ± 24	62 ± 23	57 ± 22	57 ± 28			
Men		59 ± 19	65 ± 14	59 ± 15	54 ± 11	48 ± 12			
1,25‐dihydroxyvitamin D,^1^ pmol/L									
Women	1	107 ± 47	113 ± 34	96 ± 39	102 ± 37	79 ± 20			
Men		107 ± 37	99 ± 31	96 ± 36	100 ± 39	85 ± 21			
Calcium, mmol/L									
Women	3	2.30 ± 0.10	2.30 ± 0.04	2.33 ± 0.09	2.34 ± 0.07	2.36 ± 0.07	<0.01		
Men		2.36 ± 0.11	2.38 ± 0.08	2.41 ± 0.08	2.41 ± 0.08	2.42 ± 0.11			
Ionized calcium,^2^^,^^3^ mmol/L									
Women	0	1.22 ± 0.04	1.20 ± 0.04	1.22 ± 0.03	1.22 ± 0.05	1.22 ± 0.04			<0.05
Men		1.21 ± 0.05	1.21 ± 0.04	1.22 ± 0.04	1.22 ± 0.04	1.24 ± 0.03			

Values are means ± SD.

^1^ log transformed.

^2^ Test was not performed in the UCSD study.

^3^ Ionized Ca was measured in whole blood.

Excretion of four of the measured collagen cross‐links (NTX, CTX, HP, PYD) was greater for men than for women at all time points, and excretion increased over the course of bed rest for both men and women (Table 4). The differences between sexes were eliminated when the concentrations of those markers were normalized to creatinine (data not presented), but the increase during bed rest (in men and women) remained significant. Excretion of DPD increased significantly in men but not women (Table 4).

**Table 4.** Urine markers of bone and calcium metabolism before and during different durations of bed rest

Test	Outliers, no.	Bed rest duration, days					Effect		
		0	14	30	60	90	Sex	Duration	Interaction
*n*									
Women		24	13	20	10	3			
Men		50	28	38	16	8			
N‐telopeptide,^1^ nmol/day									
Women	3	364 ± 151	493 ± 236	559 ± 250	540 ± 245	686 ± 440	<0.01	<0.001	
Men		485 ± 220	688 ± 317	809 ± 331	679 ± 244	749 ± 166			
C‐telopeptide,^2^ *μ*g/day									
Women	0	1448 ± 644	2217 ± 897	2803 ± 1287	2392 ± 785	2146 ± 37	<0.05	<0.001	
Men		2142 ± 1008	3457 ± 1944	4151 ± 1567	3948 ± 1151	4301 ± 869			
Helical peptide,^3^ *μ*g/day									
Women	3	470 ± 246	743 ± 328	727 ± 327	711 ± 257	605 ± 50	<0.01	<0.001	
Men		743 ± 385	1232 ± 596	1174 ± 522	1036 ± 228	1127 ± 229			
Pyridinium cross‐links,^1^ nmol/day									
Women	7	218 ± 64	266 ± 73	311 ± 91	301 ± 77	409 ± 139	<0.01	<0.001	
Men		285 ± 116	408 ± 148	421 ± 163	371 ± 139	389 ± 138			
Deoxypyridinoline,^1^ nmol/day									
Women	3	53 ± 16	79 ± 27	78 ± 25	81 ± 18	86 ± 22			<0.05
Men		67 ± 26	89 ± 35	100 ± 36	96 ± 29	106 ± 24			

Values are means ± SD.

^1^ log transformed.

^2^ Test was not performed in the UCSD study.

^3^ Square root transformed.

Men consumed more fluid than women (Table 5) and had greater 24‐h urine volumes. Urine volumes decreased during bed rest for both men and women (Table 6). Creatinine excretion was greater in men than women, as expected, and did not change during bed rest. Calcium excretion was greater for men than women, and increased in both sexes during bed rest (Table 6).

**Table 5.** Average intake before and during bed rest

Sex (*n*)	Before bed rest		During bed rest		Main effect		
	M (50)	W (24)	M (50)	W (24)	Sex	Bed rest	Interaction
Fluid, mL/day	4176 ± 1039	3415 ± 986	3839 ± 891	3143 ± 750	<0.01		
Calories, kcal/day	2770 ± 285	2166 ± 196	2385 ± 248	1944 ± 305	<0.001	<0.01	
Protein, g/day	106 ± 11	83 ± 8	91 ± 9	73 ± 12	<0.001	<0.01	
Calcium, mg/day	1661 ± 368	1334 ± 241	1436 ± 276	1151 ± 174	<0.001	<0.05	
Sodium, mg/day	3589 ± 400	2985 ± 345	3089 ± 376	2607 ± 532	<0.001	<0.01	
Iron,^1^ mg/day	22 ± 4	24 ± 9	19 ± 4	22 ± 8			
Phosphorus, mg/day	1795 ± 180	1429 ± 151	1534 ± 154	1246 ± 238	<0.001	<0.001	
Magnesium,^2^ mg/day	417 ± 41	327 ± 32	355 ± 38	274 ± 32	<0.001		
Vitamin D, *μ*g/day	19 ± 18	16 ± 20	19 ± 11	17 ± 11			
Methionine,^2^ g/day	2.4 ± 0.3	1.9 ± 0.1	2.1 ± 0.6	1.6 ± 0.2	<0.001	<0.01	
Cystine,^2^ g/day	1.5 ± 0.2	1.2 ± 0.1	1.3 ± 0.2	1.0 ± 0.1	<0.001	<0.001	

M, men; W, women.

^1^ *n *= 22 women.

^2^ Data were not available from the University of California San Diego study, resulting in 42 M, 17 W.

**Table 6.** Urine markers of renal stone risk before and during different durations of bed rest

Test	Outliers, no.	Bed rest duration, days					Effect		
		0	14	30	60	90	Sex	Duration	Interaction
24‐h volume, mL									
Women	0	2809 ± 848	2543 ± 710	2615 ± 606	2670 ± 649	2307 ± 394	<0.01	<0.01	
Men		3252 ± 708	2931 ± 669	3145 ± 675	3422 ± 521	3511 ± 392			
Calcium,^1^ mmol/day									
Women	5	4.5 ± 2.2	5.5 ± 2.4	5.5 ± 2.2	5.5 ± 1.3	5.4 ± 1.6	<0.05	<0.001	
Men		5.4 ± 2.1	6.6 ± 2.2	6.7 ± 2.4	6.7 ± 2.0	7.0 ± 1.5			
Oxalate,^2^^,^^3^ mg/day									
Women	3	41 ± 15	31 ± 9	36 ± 11	40 ± 14	41 ± 13			<0.05
Men		41 ± 14	39 ± 17	48 ± 17	54 ± 18	48 ± 9			
Sulfate,^3^ mmol/day									
Women	2	19 ± 2	19 ± 2	16 ± 3	15 ± 2	15 ± 2			<0.001
Men		22 ± 4	24 ± 4	23 ± 4	22 ± 4	23 ± 2			
Uric acid,^3^ mg/day									
Women	4	550 ± 107	507 ± 67	464 ± 130	404 ± 74	375 ± 101			<0.001
Men		637 ± 119	650 ± 130	589 ± 121	605 ± 127	640 ± 92			
Sodium, mmol/day									
Women	1	114 ± 29	105 ± 13	101 ± 27	92 ± 26	97 ± 18	<0.001	<0.05	
Men		139 ± 34	124 ± 21	125 ± 35	122 ± 39	131 ± 25			
Phosphorus, mg/day									
Women	8	659 ± 159	758 ± 198	661 ± 161	629 ± 122	576 ± 94	<0.001		
Men		863 ± 215	966 ± 175	901 ± 179	913 ± 210	937 ± 158			
Citrate,^3^ mg/day									
Women	1	833 ± 271	916 ± 247	705 ± 235	625 ± 167	562 ± 106	<0.01	<0.01	
Men		660 ± 253	560 ± 177	616 ± 212	612 ± 228	648 ± 224			
Magnesium,^3^ mg/day									
Women	5	96 ± 27	100 ± 31	96 ± 30	82 ± 17	92 ± 10			<0.001
Men		110 ± 33	129 ± 24	114 ± 34	115 ± 35	120 ± 29			
pH^3^									
Women	3	6.4 ± 0.3	6.4 ± 0.2	6.5 ± 0.3	6.5 ± 0.3	6.3 ± 0.2			<0.05
Men		6.5 ± 0.3	6.4 ± 0.2	6.5 ± 0.2	6.4 ± 0.3	6.5 ± 0.2			
Creatinine,^4^ mmol/day									
Women	6	4.3 ± 1.5	5.0 ± 1.9	4.4 ± 1.5	3.8 ± 0.9	4.4 ± 0.6	<0.001		
Men		5.2 ± 1.5	5.9 ± 1.6	5.6 ± 1.4	4.9 ± 0.7	5.1 ± 0.8			

^1^ Square root transformed.

^2^ Inverse transformed.

^3^ Test was not performed in the UCSD study.

^4^ log transformed.

Certain analytes including oxalate, sulfate, uric acid, and sodium contribute to an increase in renal stone risk when they are elevated. Oxalate excretion increased during bed rest for men only; sulfate and uric acid excretion decreased during bed rest for women only (Table 6). Sodium excretion decreased for both men and women during bed rest, but men had higher initial sodium excretion (and intake). The decrease in urinary sodium matched the decrease in dietary intake during bed rest (Table 5). Magnesium, pH, citrate, and phosphorus reduce renal stone risk, and each of them was found to have a unique response during these bed rest studies (Table 6). Urinary magnesium of men increased during bed rest, and urine pH of men but not women decreased. Citrate excretion decreased for men and women during bed rest, but women had higher citrate excretion before bed rest. Phosphorus excretion was greater in men before bed rest but did not change during bed rest for men or women.

Men consumed significantly more calories and more nutrients (except iron and vitamin D) than women before and during bed rest (Table 5). Total calories consumed decreased for both men and women during bed rest (Table 5). As a result of the decrease in caloric intake, nutrient intake (except magnesium) also decreased during bed rest for both men and women.

## Discussion

Bone loss during bed rest was the same for men and women, giving no indication that men and women require different considerations during selection for future space missions. Men typically have greater BMC and BMD, but the 1–3% per month whole‐body and site‐specific losses that occurred during bed rest were the same for men and women. One minor difference was that hip neck BMD decreased in women, whereas in men it was maintained or slightly increased. This result may have been caused by differing load distribution techniques during bed rest.

**Figure 2.**
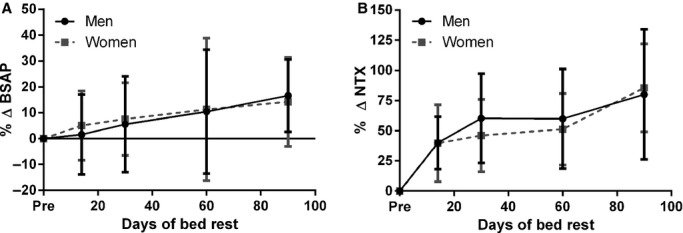
(A) Percentage change in serum bone‐specific alkaline phosphatase (BSAP) concentrations (units per liter) before and during bed rest. (B) Percentage change in urinary N‐telopeptide (NTX) concentrations (nmol per day) before and during bed rest. Gray squares and dashed lines represent data from female subjects; black circles and solid lines represent data from male subjects. Values are means ± SD.

Bone formation markers increased during bed rest, indicating that control (i.e., non‐exercising) bed rest subjects were ramping up formation of bone in response to the bone loss that was occurring because of disuse. For bed rest subjects, serum concentrations of BSAP (and ALP) were higher in men before bed rest, and in both men and women, the concentrations of these markers had increased by about 14–16% after 90 days of bed rest (Fig. 2A). Typically in bed rest studies bone formation markers remain unchanged in the control group (Smith et al. 2009b; Zwart et al. 2009b; Morgan et al. 2012b), but because of the large number of subjects in this study, we were able to detect smaller differences.

As in previous bed rest studies (Smith et al. 2005b, 2009c; Smith and Zwart 2008; Zwart et al. 2009b; Morgan et al. 2012b), the data here indicate that bone resorption increases during periods of skeletal unloading. Men and women had similar increases in bone resorption markers, with a roughly 90–107% increase measured by the end of 90 days of bed rest, relative to the concentrations of markers before bed rest (Fig. 1B). The male bed rest subjects had a systematically greater excretion of bone resorption markers for a 24‐h period. These sex differences were eliminated when the markers were normalized to creatinine (data not shown); creatinine excretion is higher for men than women because men have more muscle mass.

A few markers changed interactively with sex and bed rest. Unlike the other markers, DPD concentration was higher in women than in men (as it typically is; Gannagé‐Yared et al. 2000). During bed rest DPD showed a different interactive effect depending on whether it was normalized to excretion per day or to creatinine. The 24‐h excretion of DPD increased more for men during bed rest than for women. The concentration of DPD normalized to creatinine was higher and increased more for women than for men. These interactive effects were not observed for other biomarkers of bone resorption, suggesting that this may be an effect related to DPD.

Urine chemistry for men and women during bed rest provides some indication of renal stone risk changes that occur during periods of skeletal unloading. The responses of urine chemistry to bed rest were different for men and women. For women, about half the analytes that changed during bed rest favored lower risk of urine supersaturation while the other half favored increased risk. On the negative side, calcium excretion increased, urine volume decreased, and citrate excretion decreased for women. However, sulfate, uric acid, and sodium excretion decreased, all of which tend to reduce risk of supersaturation. The risk of urine supersaturation increases when urine volume is <2 L/day (Pak et al. 1985; Whitson et al. 2001). As the urine volumes of women throughout bed rest were more than 2.5 L/day (by design), the overall supersaturation risk for women should have been very low despite the changes in urine chemistry, and thus the overall risk was likely not changed. For men, the urine chemistry indicated that regardless of study phase, men had a higher risk of urine supersaturation than women. Before bed rest, calcium and sodium excretion were greater for men than for women, which increased the risk of renal stone formation in men from the onset of bed rest. Calcium and oxalate excretion increased, and urine pH and citrate excretion decreased during bed rest—all of which conditions are known to increase urine supersaturation. Similar to the response in women, during bed rest sodium decreased and magnesium increased for men, and both of these changes would inhibit renal stone formation. Many of these analytes may be affected by the dietary differences between men and women and the decrease in calories consumed, and not by the physiological changes that occur during bed rest. Overall, the actual risk of renal stone formation during bed rest was very low, especially for men, who were excreting more than 3 L of urine per day. If the fluid intake had been at a more typical level (e.g., <2 L/day) instead of the high volume consumed during bed rest, these changes in urine chemistry would likely have translated into an increased risk of renal stone formation for men. Recently the risk of renal stone formation was assessed in astronauts, and as these bed rest results imply, men seem to be at higher risk of stone formation than women during spaceflight (Smith et al. 2014). Reducing renal stone risk has been attempted with the use of potassium citrate supplements during spaceflight (Whitson et al. 2009) and potassium–magnesium citrate supplements during bed rest (Zerwekh et al. 2007). Potassium citrate is available on the International Space Station for crew members at high risk of renal stone formation, but fluid intake remains the primary countermeasure. Other means of reducing bone resorption, and renal stone risk, have also been tested, including pharmacologic agents and exercise protocols (Monga et al. 2006; Leblanc et al. 2013).

### Limitations of the study

It is important to note that although all of these studies were done under controlled conditions, they were not all done in the same facility, and all studies were completed over the course of several years. The bed rest condition is meant to simulate spaceflight, but certain aspects limit the extrapolation of these results to flight. One of these factors is the managed maintenance of body weight, which may alter metabolism. Additionally, the fluid intake of bed rest subjects is much greater than is typically observed in astronauts. In spite of these caveats, the study presented here contains a large sample size, providing the power to evaluate differences between men's and women's responses to bed rest. In addition, all analytes reported here were measured by the same laboratory, thereby eliminating interlaboratory variability.

## Conclusion and Implications

In summary, we document here evidence that there is little difference in bone metabolism or renal stone risk between men and women in response to bed rest. Men may be at slightly increased risk for renal stone formation as the duration of bed rest increases, but this risk is minimized in bed rest subjects with high fluid intake (2.5–3.0 L/day). These results give no indication that men and women require different considerations during selection for future space missions.

## Acknowledgments

We thank the bed rest subjects for their time and willingness to participate in this study. We thank the staff of the NASA Johnson Space Center Nutritional Biochemistry Laboratory for their assistance in processing and analyzing the samples, and in all aspects of carrying out this project. We thank the NASA JSC Immunology/Biochemical Analysis Lab for assistance with the renal stone profile analyses. We thank Jane Krauhs for editorial assistance.

## Conflict of Interest

None declared.
